# Building the Evidence for Advance Care Planning for Patients Receiving Dialysis

**DOI:** 10.1001/jamanetworkopen.2023.52415

**Published:** 2024-01-02

**Authors:** Manjula Kurella Tamura, Laura M. Holdsworth

**Affiliations:** Division of Nephrology, Stanford University School of Medicine, Palo Alto, California; Geriatric Research and Education Clinical Center, Palo Alto VA Health Care System, Palo Alto, California; Division of Primary Care and Population Health, Stanford University School of Medicine, Stanford, California

Due to advances in multiple care domains, the survival of patients who receive long-term dialysis for kidney failure has improved over the past 2 decades.^1,2^ Success has its price, however. In the case of kidney failure, improvements in dialysis survival, coupled with an aging population and limited supply of transplant organs, mean that more patients than ever before are living and dying with kidney failure while receiving dialysis therapy.

There is ample evidence that patients receiving dialysis and their surrogates are not prepared for the complex set of decisions they face as they approach the end of life. As a consequence, they receive high-intensity care at the end of life that is potentially unwanted and nonbeneficial.^3,4^ The value of timely, high-quality advance care planning (ACP) before a health care crisis in this population seems obvious. Done well, ACP facilitates understanding each patient’s priorities for living with serious illness and enables patients to plan for future health care decisions. Yet, the promise of ACP has fallen short of its potential, partly due to limited implementation in dialysis practice and ambivalence from clinicians about ACP’s effectiveness. Studies show that only half of patients receiving dialysis have engaged in discussions about treatment preferences, and fewer than 1 in 3 have documented their preferences and identified a surrogate decision-maker.^2^

Central to effectively implementing a complex practice, such as ACP, is ensuring that the intervention modality and approach are appropriate for the population and context. For example, ACP delivered by clinicians with special training in goals-of-care conversations may be highly efficacious but not scalable and, therefore, have limited impact. An ACP intervention targeted to patients with the highest mortality risk might make the most of limited clinician time. However, this implementation approach runs the risk of missing patients who could benefit from ACP, and it may provoke anxiety if patients feel singled out for the practice.

In this issue of *JAMA Network Open*, Song and colleagues^5^ test the effectiveness of an ACP intervention, Sharing the Patient’s Illness Representations to Increase Trust (SPIRIT), when delivered at scale in a large and diverse population of patients receiving dialysis. The trial enrolled 426 patient-surrogate dyads from 42 freestanding dialysis clinics in 5 states. Dyads were randomized at the clinic level to usual ACP care, consisting of written information about advance directives from the clinic social worker, vs the SPIRIT ACP intervention. Developed over a series of pilot and efficacy trials, the SPIRIT intervention consists of a guided discussion of the patient’s illness experience and values concerning treatments at the end of life. SPIRIT aims to enable patients to set goals and prepare them and their surrogates for the emotional burden of end-of-life decision-making. SPIRIT was delivered during a single session, with an option for a follow-up session 2 weeks later. Each clinic designated 1 or 2 SPIRIT champions who led all SPIRIT sessions after undergoing communication skills training.

The reach and uptake of ACP in the trial were impressive: half of all eligible patients enrolled in the trial, and approximately three-fourths of the patient-surrogate dyads assigned to the SPIRIT group completed the second ACP session. Compared with control dyads who received usual ACP care, dyads randomized to SPIRIT had more congruence in goals of care and less decisional conflict at 2 weeks. In addition, dyads receiving SPIRIT experienced an improvement in a composite outcome of dyad congruence and surrogate decision-making confidence. Bereavement outcomes were measured in a subset of 77 surrogates who experienced the death of their loved one after enrollment. Compared with surrogates who received usual ACP care, surrogates who received SPIRIT experienced lower levels of bereavement anxiety. In contrast to the prior efficacy trials, however, SPIRIT did not reduce symptoms of depression or posttraumatic stress.

The authors posit SPIRIT’s effects on bereavement outcomes were less robust than in prior efficacy studies despite apparently high intervention fidelity due to distress caused by the COVID-19 pandemic. A post hoc analysis of bereavement outcomes stratified by timing of assessment (pre- vs postpandemic) provides support for this hypothesis, but the small sample precludes a definitive conclusion. A separate possibility is that the fidelity of conversations may have waned after trial activities were interrupted by the pandemic, and pandemic fatigue set in.

The study by Song et al^5^ is one of the largest trials of ACP in a population of patients receiving dialysis. Like all important studies, it leaves some unanswered questions. To reach a broader population, pragmatic trials often make trade-offs in intervention flexibility and a more limited set of outcomes. Here, the investigators measured the primary outcome at 2 weeks, when retention of the ACP session was likely at its highest but not at later time points. In addition, the study did not measure whether the ACP intervention resulted in less intensive treatment or more goal-concordant care. Also, though the study examined fidelity, additional implementation outcomes such as sustainability and costs were not expressly included in the current report. These outcomes would be informative for understanding strategies needed for implementation.

The trial by Song and colleagues^5^ offers some important lessons for implementing serious illness models of care in kidney failure. First, SPIRIT’s robust reach and uptake speaks to patients’ interest and desire for context-appropriate ACP. Second, the trial supports leveraging multidisciplinary team roles, relieving the bottleneck caused by limited physician time. Indeed, it demonstrates that 1 or 2 people per clinic trained in serious illness conversation can affect patient and surrogate outcomes. Third, the trial extends our understanding of the value of ACP to patients receiving dialysis. The psychological benefits of ACP for patients, families, and clinical teams appear consistent across settings and populations.^6^ We have found that clinicians participating in ACP see benefits beyond those easily quantified, such as deepening the patient-clinician relationship and improving confidence to discuss sensitive topics.^7^ Additionally, by demonstrating the effectiveness of an ACP intervention on patient-centered outcomes, the trial provides preliminary support for a dialysis quality framework anchored to goals that matter to individual patients, a concept known as goal-directed dialysis. This idea represents a marked departure from population-level quality programs currently used in dialysis that may inadvertently limit patient choice.^8,9^

Song and colleagues^5^ are to be commended for conducting a high-quality trial of ACP in this complex population and setting. While much is still to be learned, the study is an important building block in the evidence base for patient-centered dialysis care.
